# A predictive Bayesian network that risk stratifies patients undergoing Barrett’s surveillance for personalized risk of developing malignancy

**DOI:** 10.1371/journal.pone.0240620

**Published:** 2020-10-12

**Authors:** Alison Bradley, Sharukh Sami, Hwei N. G., Anne Macleod, Manju Prasanth, Muneeb Zafar, Niroshini Hemadasa, Gregg Neagle, Isobelle Rosindell, Jeyakumar Apollos

**Affiliations:** Department of General Surgery, Dumfries and Galloway Royal Infirmary, NHS Dumfries and Galloway, Dumfries, Scotland, United Kingdom; Baylor College of Medicine, UNITED STATES

## Abstract

**Background:**

Barrett’s esophagus is strongly associated with esophageal adenocarcinoma. Considering costs and risks associated with invasive surveillance endoscopies better methods of risk stratification are required to assist decision-making and move toward more personalised tailoring of Barrett’s surveillance.

**Methods:**

A Bayesian network was created by synthesizing data from published studies analysing risk factors for developing adenocarcinoma in Barrett’s oesophagus through a two-stage weighting process.

**Results:**

Data was synthesized from 114 studies (n = 394,827) to create the Bayesian network, which was validated against a prospectively maintained institutional database (n = 571). Version 1 contained 10 variables (dysplasia, gender, age, Barrett’s segment length, statin use, proton pump inhibitor use, BMI, smoking, aspirin and NSAID use) and achieved AUC of 0.61. Version 2 contained 4 variables with the strongest evidence of association with the development of adenocarcinoma in Barrett’s (dysplasia, gender, age, Barrett’s segment length) and achieved an AUC 0.90.

**Conclusion:**

This Bayesian network is unique in the way it utilizes published data to translate the existing empirical evidence surrounding the risk of developing adenocarcinoma in Barrett’s esophagus to make personalized risk predictions. Further work is required but this tool marks a vital step towards delivering a more personalized approach to Barrett’s surveillance.

## Introduction

The incidence of esophageal malignancy has increased markedly within the Western World in recent decades [1, 2]. Most cases of esophageal malignancy can be attributed to known risk factors including gastro-esophageal reflux disease, smoking, central obesity and hiatus hernia. Family history, male gender and Caucasian race have also been linked to increased risk of developing esophageal malignancy [1].

Barrett’s esophagus has a 30 to 125 times greater risk of developing esophageal adenocarcinoma compared to age matched population [2–4]. Barrett’s surveillance screening has therefore been recommended as this has been shown to detect disease at an earlier stage resulting in prolonged survival times. However, the relative rarity of malignancy balanced against the invasiveness of upper gastrointestinal endoscopy, has meant that the cost-effectiveness of Barrett’s surveillance depends on the risk of cancer with a wide variation in this risk being observed and reported [1, 2]. Definitions of Barrett’s esophagus, clinical guidelines and criteria for screening also vary between countries and professional bodies [1].

The challenge is to identify at individual patient level who will benefit most from surveillance endoscopy and to what intensity of frequency this should be performed in order to maximize benefit and minimise both risk and costs of the procedure itself as well as risk of failure to detect an early malignancy [1, 2]. The second challenge therefore is how we can better communicate and transmit complex narratives to patients about their individual risk over time following a diagnosis of Barrett’s esophagus to facilitate better shared decision-making.

Personalized predictive modeling has gained precedence within contemporary medicine [5, 6]. However existing predictive models are rarely applied in clinical practice as they are mainly based on non-linear regression techniques and fail to capture the dynamic nature of the care process whereby predicted outcomes evolve as time-dependent information emerges [7, 8]. Bayesian networks offer a means of engaging with the complexity of a healthcare process.

The aim of this study is to create a Bayesian network that provides patients with Barrett’s esophagus with individualised risk assessments for developing high-grade dysplasia or adenocarcinoma. We hypothesize that by using a Bayesian network to model risk as a complex adaptive system, we will create a model that can provide more accurate individualized predictions of risk throughout the patient journey, with predictive updating as patient information evolves over time, compared to relying on degree of dysplasia alone.

## Materials and methods

### Bayesian network

Bayesian networks are graphical models that are based on probability theory [9]. They are also referred to as acyclic directed graphs and model the relationship between variables, or nodes, with arcs depicting causal relationships between parent and child nodes based on a joint or multivariate probability distribution [9, 10]. This means that each node within the Bayesian network has a defined and exclusive set of states [11]. Through Bayes theorem the state of a child node is defined by the condition of its parent nodes, with the dependencies between parent and child nodes quantified through a set of conditional probability tables, which has been proven to be an effectively method of handling uncertainty within a model [11–13].

This is often viewed as the likelihood distribution and in a Bayesian network this can be computed as [9]:

$$p(Xi|Y)=\frac{p(Y|Xi)xp(Xi)}{\Sigma jp(Y|Xj)p(Xj)}$$

where *X*_*i*_ is any mutually exclusive parameter, _(*i* = 1,2,3…n)_, given the observed state of *Y*. *p*(*X*|*Y*) is the posterior probability of *X* given the condition of *Y*, and *p*(*Y*|*X*) is the posterior probability of *Y* given the condition of *X*, with the prior probability of *X* represented by *p*(*X*) and the marginal occurrence of *Y* presented *p*(*Y*) [9].

Bayes theorem therefore allows the prior distribution and observed data to be combined to update knowledge in the form of the posterior distribution which in practical terms allows predictions to evolve as more information becomes available at different stages of the healthcare process [6, 9, 14, 15]. Bayesian networks can therefore also perform diagnostic and decision analysis through marginalization, which is also employed to compute the reliability of networks based on statistical data and allows clinicians to assess ‘what if’ scenarios [16–18].

### Evidence synthesis

Conditional probabilities used within a Bayesian network can, in some circumstances, be elicited from expert opinion but this can result in larger, more complex models losing reliability [10, 11, 19]. An alternative approach for acquiring conditional probabilities could be through the use of training data [11]. However, the risk of cancer in Barrett’s patients has been reported as below 3 per cent per annum and the lack of consistency in definitions and surveillance guidelines as well as the lack of national surveillance databases in some countries makes the acquisition of sufficiently large and detailed databases difficult [1, 2]. This has resulted in previous prediction models being limited by small and/or biased datasets and lacking generalizability. Methods first developed by Zhao and Weng [20], and later adapted by Bradley *et al*. [21], were therefore employed to synthesize evidence from which to build the Bayesian network.

First PubMed, Cochrane Database, and GoogleScholar databases were searched following the PRISMA guidelines [22] (S1 Fig) with the entire database included from 1^st^ January 2000 up to and including 23^rd^ December, 2019 using the full list of search terms provided in (S1 Table). The inclusion criteria were full-text multivariable analysis studies of risk factors for developing high-grade dysplasia or esophageal malignancy in patients aged 18years or over who had Barrett’s esophagus. Observational and cohort studies and studies that reported only rates or incidence of malignancy without multivariable analysis of variables associated with its development were excluded. Studies exploring experimental treatment strategies or comparing surveillance strategies were excluded. Reference lists and citations of all included papers were manually screened to identify any additional articles until no new articles were identified.

The first and second author performed search design and all authors performed independent data extraction and quality assurance with any discrepancies resolved by inter-reviewer discussion. Data was extracted manually from studies and included: study year, number of included patients, all variables that were included in the multivariable analysis, and for each variable whether it was found to have a statistically significant association with the development of high-grade dysplasia or malignancy as defined by a P value <0.005. This yielded 114 papers, (n = 394,827) from which the model was built (S2 Table).

Secondly extracted data underwent a two stage weighting process developed by Zhao and Weng [20], and developed further by the work of Bradley *et al*. [21]. The original weight for each variable represented a summary of existing evidence, including conflicting findings [20]. The original weight then underwent a process of normalization to place this ratio in the context of the entire body of evidence related to each variable (Table 1; S3 Table). This approach has been shown to reduce the impact of bias inherent in the literature on the model as conflicting findings are accounted for and smaller trials with greater risk of bias have a reduced impact through the process of normalization [20, 21].

**Table 1:** Top 10 weighted variables from synthesized studies ordered in rank order based on normalized weighting.

Variable/ Node	Node States within Bayesian Network
Dysplasia	No dysplasia
	Low grade dysplasia
	High grade dysplasia
Gender	Female
	Male
Age	<60 years
	60-70year
	>70 years
Segment Length	<3cm
	3cm-5cm
	>5cm
Statin	Yes
	No
Proton Pump Inhibitor (PPI) use	Yes
	No
Body Mass Index (BMI)	>18/<28
	28–30
	>30/<18
Smoking	No
	Ex/Current
Aspirin	Yes
	No
Non-Steroidal Anti-Inflammatory (NSAID)	Yes
	No

### Bayesian network structure

**Fig 1:**
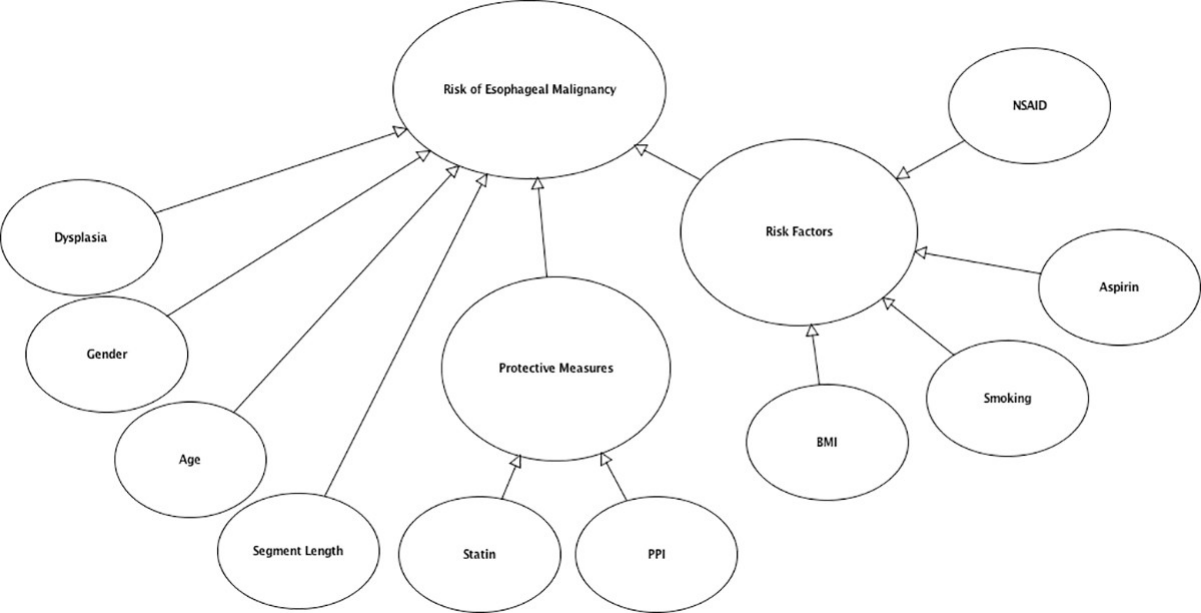
Bayesian network version 1 based on top 10 variables.

**Fig 2:**
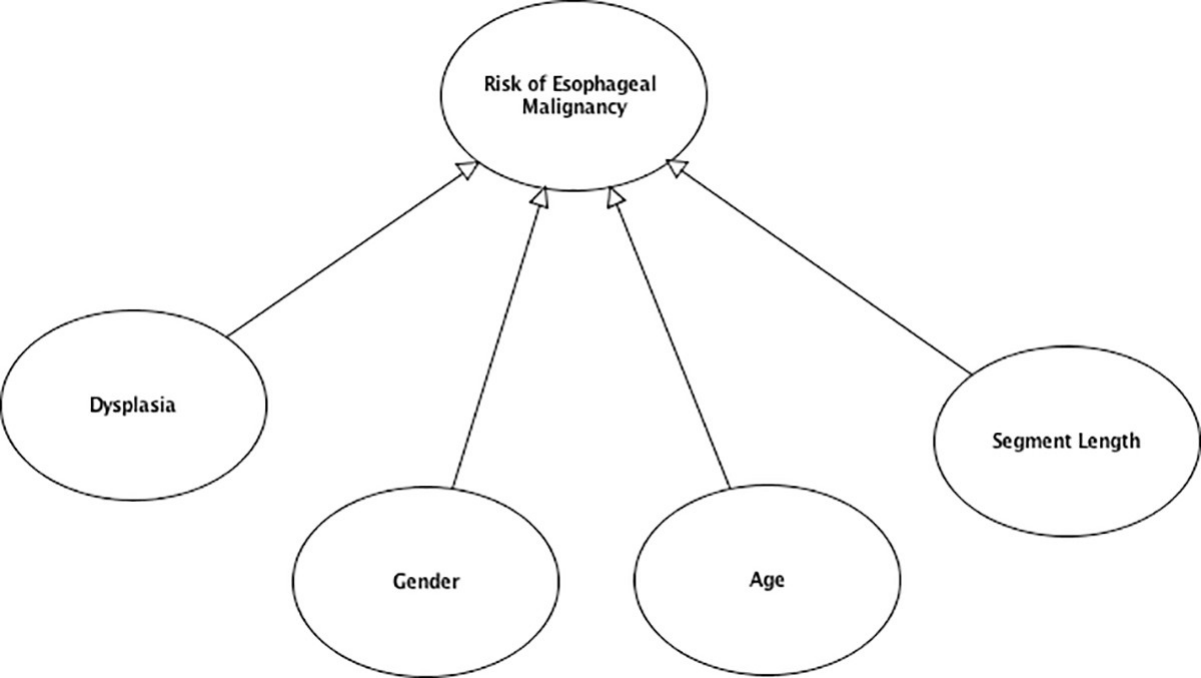
Bayesian network version 2 based on top 4 variables.

The top 10 ranking variables (Table 1) were used to structure the first version of the Bayesian network created using AgenaRisk version 10 software [23] (Fig 1). The definitions and categorization of input data for each node detailed in Table 1 were determined by how this data was presented in published studies and were approved by an expert panel of surgeons. Continuous variables such as age and length of Barrett’s segment were therefore modeled as categorical variables based on how these variables were reported in published studies (Table 1). Node probability tables for each child node was calculated using the normalized weighting of each parent node as the weighted mean of the truncated Normal (TNormal) distribution which has been proven to generate accurate node probability tables for Bayesian networks involving ranked nodes with ranked parent nodes [14]. The second version of the Bayesian network was based on only the top 4 ranked variables based on their normalized weighting. These nodes acted as direct parent nodes of the output node (Fig 2). The output node was calculated from the weighted mean of the corresponding parent nodes. The output node provided a percentage probability of having low, medium or high risk of developing high-grade dysplasia or esophageal malignancy.

### Model validation

**Fig 3:**
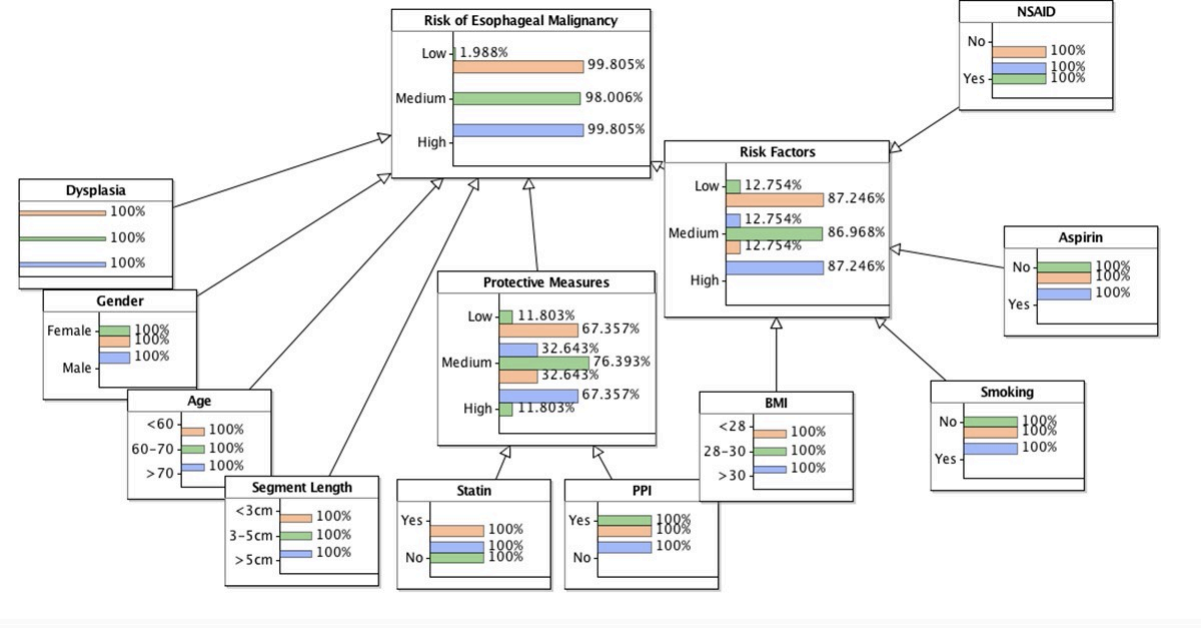
Bayesian network version 1 based on top 10 variables. Scenario 1 blue; scenario 2 green; scenario 3 orange.

**Fig 4:**
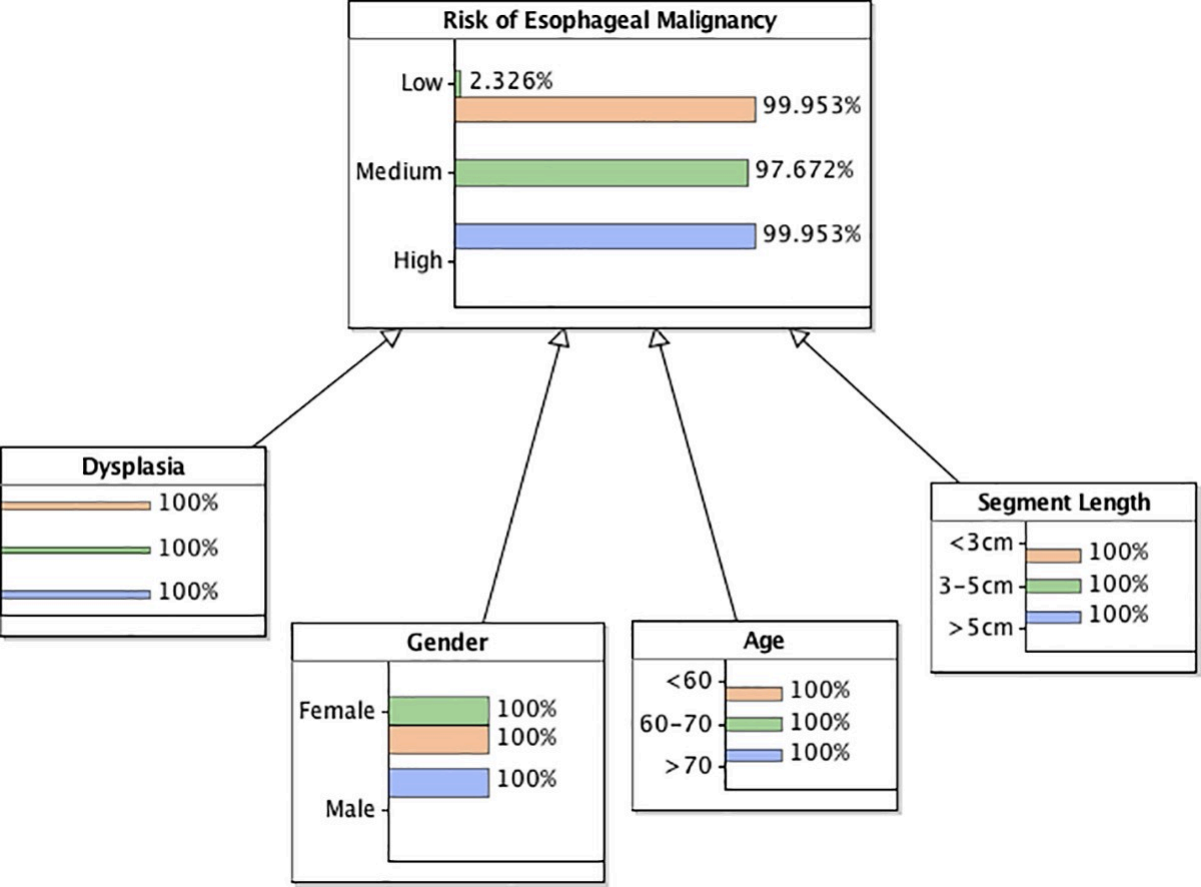
Bayesian network version 2 based on top 4 variables. Scenario 1 blue; scenario 2 green; scenario 3 orange.

The proposed Bayesian networks were checked with three hypothetical scenarios. Scenario 1 reflects a high-risk patient where all the criteria are in worst possible state. Scenario 2 reflects a patient where all criteria are in medium or moderate states. Scenario 3 reflects a low-risk patient where all criteria are in the most favourable state (Figs 3 and 4).

The performance of the model was assessed using the area under the curve (AUC) of the receiver operating curve (ROC) using SPSS Statistics version 26 software. It was validated against the prospectively maintained Barrett’s surveillance patient database of a district general hospital. This database contained 571 patients and two expert pathologists confirmed all pathology reports. Anonymous individual patient data for all 517 patients was entered into the Bayesian network to provide individual patient risk assessment of developing high-grade dysplasia or esophageal malignancy for each patient. On completion of the model all patients within the database were followed up. 62 were due and had undergone their repeat upper gastrointestinal surveillance endoscopy or had been diagnosed with malignancy and undergone definitive treatment. The original risk profile of these patients were compared against the results from their repeat surveillance endoscopy to validate the performance of the Bayesian network. Those patients who had an initial predicted high risk of malignancy of 50% or above were expected to have developed high-grade dysplasia or malignancy for the Bayesian network to make a true positive prediction.

## Results

There were 54 patients who had repeat endoscopy or definitive treatment and who had complete data on all 10 variables contained within version 1 of the Bayesian network. Validated against these patients version 1 of the Bayesian network had a sensitivity of 0.55 and specificity of 1 with a positive predictive value of 1 and negative predictive value of 0.90. AUC was 0.61 (standard error 0.33; P value 0.001; 95% confidence interval 0.000–0.126).

**Fig 5:**
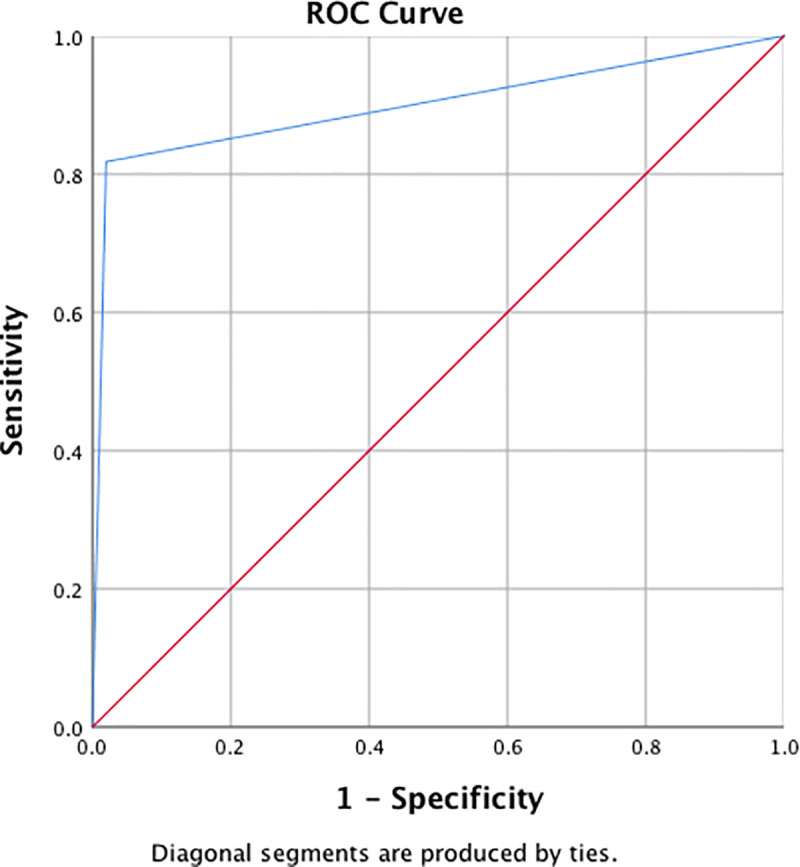
Bayesian network version 2 area under receiver operator curve.

Version 2 of the Bayesian network was based on only the 4 variables most strongly associated with developing esophageal malignancy and performed better than version 1. There were 62 patients who had repeat endoscopy or definitive treatment and who had complete data on all 4 variables contained within version 2 of the Bayesian network. Validated against these patients version 2 of the Bayesian network had a sensitivity of 0.82 and specificity of 0.98 with a positive predictive value of 0.90 and negative predictive value of 0.96. AUC was 0.90 (standard error 0.07; P value 0.000; 95% confidence interval 0.762–1.000) (Fig 5). When only dysplasia was used within the Bayesian network this produced a sensitivity of 1.0 and specificity of 0.79 with a positive predictive value of 0.36 and negative predictive value of 0.84. AUC was 0.68 (standard error 0.10; P value 0.060; 95% confidence interval 0.477–0.886).

## Discussion

This Bayesian network was developed to provide patients with Barrett’s esophagus with an individualised risk assessment for developing esophageal adenocarcinoma. Such information could facilitate better shared decision-making regarding the potential impact of preventative measures and decisions regarding the intensity of surveillance. By utilizing existing published data in a unique way within a Bayesian network, version 2 of the model was able to achieve an AUC of 0.90, which outperformed reliance on dysplasia alone.

The evidence base for predicting progression to esophageal adenocarcinoma in Barrett’s esophagus is limited which makes comparison of this model’s performance difficult [1]. Previous studies assessing the relative risk of malignancy in this population are mainly small cohort studies which lack generalizability, have limited and often conflicting risk factor information, focus on a small number of outcomes for a relatively rare occurrence rate of esophageal malignancy and use varying study designs [1]. Across existing studies there is disagreement about what factors are significantly predictive and not all studies include data on all known potential factors affecting risk [1].

### Strengths and limitations

This model is unique in its novel utilization of knowledge from existing studies, including the incorporation of conflicting information and uncertainty across studies through a two stage weighting process. This means that the existing evidence base can be translated in a clinically more meaningful way for individual patients and their clinicians to assist shared decision-making. This also means that the Bayesian network overcomes the limitations of many existing models such as lack of generalizability and bias from being largely based on single institutional databases.

A limitation of this model is that it is based on published studies that are mainly cohort analysis from single centres that did not explicitly state whether pathology reports were confirmed by two pathologists, and which demonstrated a degree heterogeneity in surveillance strategies, which carries a risk of bias. The two-stage weighting process of variables was designed to minimize the potential impact of such bias on the Bayesian network [21]. There is also the potential that new and emerging studies will alter the weightings of nodes within the model. A strength of the Bayesian network is that new advances either in less invasive methods of surveillance of in our understanding of disease at molecular or genetic level can easily be incorporated within the model which effectively offers a vehicle to combine clinical and genomic data in a clinically meaningful way [21]. Thus far the model has only been prospectively validated against a small number of patients. Further prospective validation is ongoing and the model would also benefit from being validated against another institution’s database. The anticipated next phase of this model will be to incorporate patient level data from larger national databases into the existing model so that the accuracy of predictions can be further improved by combining the prior distribution and observed data to update the posterior distribution through Bayes theorem [14].

### Study impact

**Fig 6:**
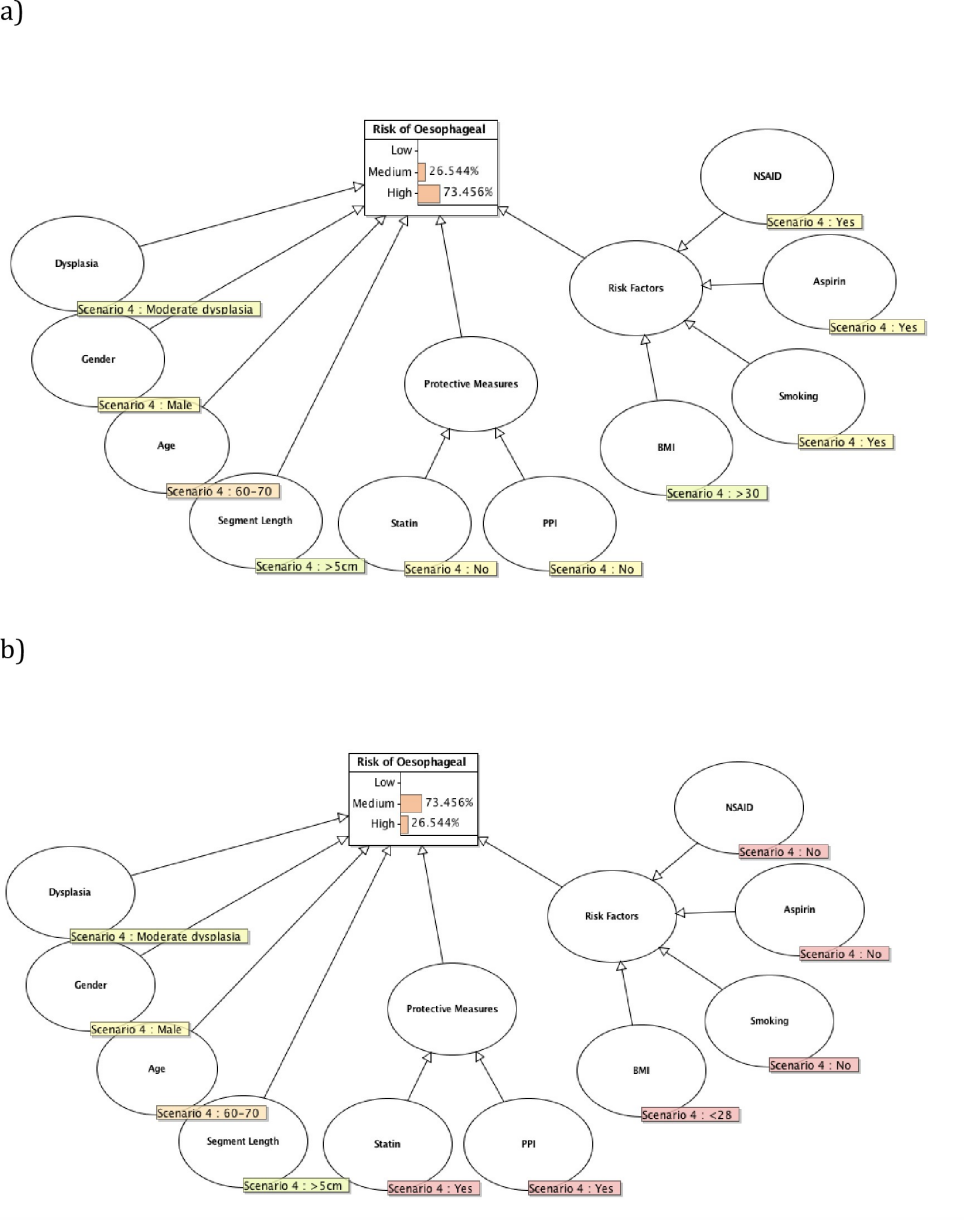
Bayesian network showing how the individual patient’s risk profile alters without and then with implementation of protective measures.

Barrett’s esophagus is strongly associated with esophageal malignancy. However there are significant costs and risks associated with invasive surveillance endoscopies. Ambiguities also exist in the definitions of Barrett’s esophagus, clinical guidelines and criteria for screening between countries and professional bodies [1]. Current surveillance strategies rely heavily on degree of dysplasia and the Bayesian network presented here weights this variable as holding strongest significance within the model. This study builds on previous work by Bradley *et al*. [21] to model under uncertainty to move towards a more personalized approach to risk stratification in order to assist clinical decision-making. In the immediate clinical setting the potential impact of this study could be to help to communicate a complex narrative to patients regarding their individual risk of malignancy, as it evolves over time, following a diagnosis of Barrett’s esophagus to facilitate better shared decision-making [21]. Both versions of the Bayesian network presented here go beyond a reliance solely on dysplasia. The AUC for version 2 of the model is superior to that produced by modeling dysplasia alone. Version 1 of the Bayesian network also offers additional benefits and insights that facilitate better communication and shared decision-making in the clinical setting that a reliance on dysplasia alone could not offer. This includes being better able to explain to patients the impact of “what if” scenarios on their individual risk profiles such as the impact of risk reduction strategies including weight loss in obese patients, smoking cessation and the introduction of proton pump inhibitors et cetera, as demonstrated in Fig 6. Therefore whilst version 2 of the model might be used to make more accurate predictions, version 1 of the Bayesian network still has clinical merit in facilitating better clinical communication and shared decision-making through graphical representation of individualized risk profiles.

A further impact of this research could be in directing future research towards developing more sophisticated mechanisms of clinical decision support and analysis models to support the delivery of more personalized medicine through the integration of multiple, large and complex databases [21, 24–27]. The methods used and developed within this study have the potential to merge clinical and pathological data to make individualized predictions of risk of developing esophageal malignancy in patients with Barrett’s esophagus. At present American College of Gastroenterology guidelines state that biomarkers should not be used for surveillance [28]. However, as our knowledge of disease processes evolve to include a deeper genetic understanding, a future requirement will emerge to develop ways of amalgamating both genetic and clinical data in order to facilitate better shared clinical decision-making. As demonstrated in this study Bayesian networks offer one possible solution. As newer methods of Barrett’s screening are developed, and our understanding of disease at molecular level advances, this emerging data could be integrated into this model as additional weighted nodes, with the posterior data distributions updated accordingly through Bayes theorem, hence facilitating the clinical application of such knowledge at individual patient level [21, 27].

## Supporting information

S1 Fig PRISMA flowchart. (TIF)

S1 Table Search terms. (DOCX)

S2 Table Summary of included studies. (XLSX)

S3 Table Variables ranked. (XLSX)
