# Supplementary figures and images for: A predictive Bayesian network that risk stratifies patients undergoing Barrett’s surveillance for personalized risk of developing malignancy
Source: PLoS One. 2020 Oct 12;15(10):e0240620. doi: 10.1371/journal.pone.0240620 (PMC7549831; doi:10.1371/journal.pone.0240620)

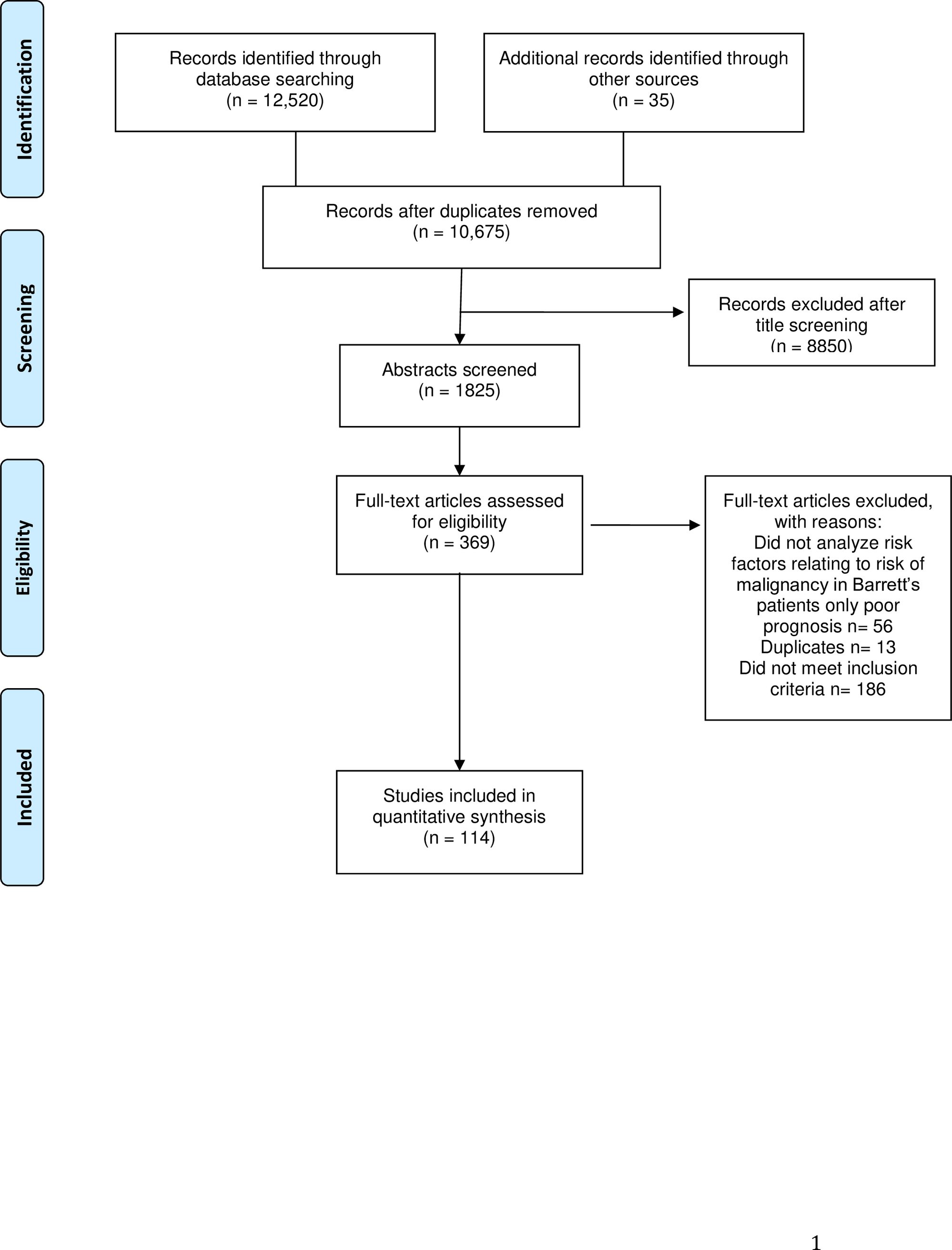

Supplement: S1 Fig — (TIF) [file pone.0240620.s001.tif]
