# Supplementary material for: A predictive Bayesian network that risk stratifies patients undergoing Barrett’s surveillance for personalized risk of developing malignancy
Source: PLoS One. 2020 Oct 12;15(10):e0240620. doi: 10.1371/journal.pone.0240620 (PMC7549831; doi:10.1371/journal.pone.0240620)
Supplement: S1 Table — (DOCX) [file pone.0240620.s002.docx]

| Search Terms Used |
| --- |
| Barrett's[All Fields] OR "barrett esophagus"[MeSH Terms] OR "esophageal neoplasms"[MeSH Terms] AND "risk factors"[MeSH Terms] OR risk factors[Text Word] OR prediction[All Fields] OR "risk assessment"[MeSH Terms] OR risk assessment[Text Word] OR predictive[All Fields] |
